# The effect of volume of interest definition on quantification of lymph node immune response to a monkeypox virus infection assessed by ^18^F-FDG-PET

**DOI:** 10.1186/s13550-014-0049-z

**Published:** 2014-09-16

**Authors:** Svetlana Chefer, Richard C Reba, Christopher Z Leyson, Jurgen Seidel, Reed F Johnson, Joseph E Blaney, Peter B Jahrling, Julie Dyall

**Affiliations:** Division of Clinical Research, Integrated Research Facility, National Institute of Allergy and Infectious Diseases, National Institutes of Health, 8200 Research Plaza, Frederick, MD 21702 USA; Center for Infectious Disease Imaging, Radiology and Imaging Sciences, Clinical Center, National Institutes of Health, 10 Center Drive, Bethesda, MD 20892 USA; Division of Intramural Research, Emerging Viral Pathogens Section, National Institute of Allergy and Infectious Diseases, National Institutes of Health, 8200 Research Plaza, Frederick, MD 21702 USA

**Keywords:** PET, Immune response quantitation, Animal model, SUV, Monkeypox, Intravenous inoculation

## Abstract

**Background:**

2-deoxy-2-[^18^F]fluoro-D-glucose-positron emission tomography (^18^F-FDG-PET) is applied in the clinic for infection assessment and is under consideration for investigating the inflammatory/immune response in lymphoid tissue in animal models of viral infection. Assessing changes in ^18^F-FDG uptake of lymph nodes (LNs), primary lymphoid tissues targeted during viral infection, requires suitable methods for image analysis. Similar to tumor evaluation, reliable quantitation of the LN function via multiple ^18^F-FDG-PET sessions will depend how the volume of interest is defined. Volume of interest definition has a direct effect on statistical outcome. The current study objective is to compare for the first time agreement between conventional and modified VOI metrics to determine which method(s) provide(s) reproducible standardized uptake values (SUVs) for ^18^F-FDG uptake in the LN of rhesus macaques.

**Methods:**

Multiple ^18^F-FDG-PET images of LNs in macaques were acquired prior to and after monkeypox virus intravenous inoculation. We compared five image analysis approaches, SUV_max_, SUV_mean_, SUV_threshold_, modified SUV_threshold_, and SUV_fixed volume_, to investigate the impact of these approaches on quantification of the changes in LN metabolic activity denoting the immune response during viral infection progression.

**Results:**

The lowest data repeatability was observed with SUV_max_. The best correspondence was between SUV_fixed volume_ and conventional and modified SUV_threshold_. A statistically significant difference in the LN ^18^F-FDG uptake between surviving and moribund animals was shown using modified SUV_threshold_ and SUV_fixed volume_ (adjusted *p* = 0.0037 and *p* = 0.0001, respectively).

**Conclusions:**

Quantification of the LN ^18^F-FDG uptake is highly sensitive to the method applied for PET image analysis. SUV_fixed volume_ and modified SUV_threshold_ demonstrate better reproducibility for SUV estimates than SUV_max_, SUV_mean_, and SUV_threshold_. SUV_fixed volume_ and modified SUV_threshold_ are capable of distinguishing between groups with different disease outcomes. Therefore, these methods are the preferred approaches for evaluating the LN function during viral infection by ^18^F-FDG-PET. Validation of multiple approaches is necessary to choose a suitable method to monitor changes in LN metabolic activity during progression of viral infection.

**Electronic supplementary material:**

The online version of this article (doi:10.1186/s13550-014-0049-z) contains supplementary material, which is available to authorized users.

## Background

Assessment of cell glucose metabolism by 2-deoxy-2-[^18^F]fluoro-D-glucose (^18^F-FDG) and positron emission tomography (PET) is a powerful supplement to conventional studies of viral infection in animal models to characterize disease progression and evaluate the efficacy of potential treatments [1,2]. Lymphadenopathy is one of the predominant clinical signs of monkeypox virus infection in nonhuman primates (NHPs) and humans. Therefore, a reasonable method to monitor the evolving lymph node (LN) immune response is assessment of LN metabolic activity using standardized uptake value (SUV) as a simple semiquantitative measure of ^18^F-FDG uptake [3,4]. Similar to the methodological issues associated with the analysis of ^18^F-FDG-PET images in oncology [5], computing the SUVs and reliably evaluating the LN immune response to viral infection will depend on an exact and reproducible definition of the volume of interest (VOI). VOI definition for PET image quantitation is still an open research area, and users are applying the most reliable and reproducible techniques suitable for analysis of different types of disease (e.g., tumors, inflammatory conditions). Although the VOI definition is not the only factor that can affect the reproducibility of SUV estimates in the LN [5-7], the type and size of a VOI may greatly contribute to the variability of such measurements. Such variation has been previously demonstrated with tumor quantitation using ^18^F-FDG-PET imaging [7,8].

A variety of methods have been proposed to define tumor VOI, but no reference standard has been accepted. Commonly used approaches for quantitative analysis of ^18^F-FDG-PET images include the following: 1) measuring the value of the voxel with the highest activity within the tumor (SUV_max_) [9,10], 2) averaging the SUVs from the voxels inside the whole tumor defined by freehand outline of tumor boundaries (SUV_mean_) [11-13], 3) averaging the voxels with the SUVs greater than a certain percentage of SUV_max_ using thresholding techniques (SUV_threshold_) [7,14,15], or 4) using fixed volume (SUV_fixed volume_) defined as the average SUV within a fixed-size VOI centered over a region with high metabolic activity without conforming to the precise tumor outline. A similar concept of SUV_fixed volume_ has been used by Boellaard et al. and was called SUV_peak_ [6].

These VOI metrics are rather general and, as such, are not optimized to detect reproducible changes in ^18^F-FDG uptake (as measured by SUVs) during viral infection progression in a small target organ (LN). In particular, ^18^F-FDG uptake during early viral infection is difficult to measure as normal or near normal LNs have low glycolytic activity comparable to background. The lack of data on the agreement between varied methods to provide reproducible SUV estimates of the metabolic activity of LN has prompted us to develop new methods for VOI metrics. We evaluated new methods, SUV_fixed volume_ and modified SUV_threshold_ (mSUV_threshold_), against conventional (SUV_max_, SUV_mean_, SUV_threshold_) metrics. Here, we report the statistical reliability of each of these methods on quantitative assessment of ^18^F-FDG uptake changes in axillary LNs of rhesus macaques (*Macaca mulatta*) following a monkeypox virus intravenous challenge. To test interscan reproducibility, data from three baseline computed tomography (CT) and PET scans prior to monkeypox virus inoculation were used to measure LN volumes and SUV_max_, SUV_mean_, SUV_threshold_, SUV_fixed volume_, and mSUV_threshold_ in these animals.

## Methods

Animals were housed in a facility accredited by the Association for Assessment and Accreditation of Laboratory Animal Care International. All experimental procedures were approved by the National Institute of Allergy and Infectious Diseases, Division of Intramural Research, Animal Care and Use Committee and were in compliance with the Animal Welfare Act regulations, Public Health Service policy, and the *Guide for the Care and Use of Laboratory Animals* recommendations.

### Subjects

Six male rhesus macaques housed in biosafety level 3 containment, weighing 3 to 4 kg, were infected intravenously with 5 × 10^7^ plaque forming units of monkeypox virus (MPXV Zaire 79 strain [V-79-I-005]) (for virus preparation and inoculation procedures, see Additional file 1). Three animals were treated intravenously with cidofovir (5 mg/ml/kg in Dulbecco’s modified Eagle’s medium; Gilead Sciences, Foster City, CA, USA) that has been shown to protect against monkeypox virus infection. The antiviral agent, cidofovir, was administered on day −1 prior to monkeypox virus challenge and on days +1, +3, +5, +7, +10, and +13 after challenge. NHPs received 25 mg/kg of probenecid by gavage 1 h before cidofovir injection to prevent cidofovir nephrotoxicity. Three animals comprised the untreated control group.

### Data acquisition

Up to nine imaging sessions were conducted in each of the six animals following the procedures described previously [1]. Briefly, imaging data were acquired in animals anesthetized with isoflurane (2% to 2.5%) (Piramal Critical Care, Orchard Park, NY, USA) using a microPET scanner Focus-220 (Siemens AG, Malvern, PA, USA). This scanner has a bore size of 22 cm with an axial field-of-view of 7.6 cm and a transverse field-of-view of 19 cm [16]. Multiple static PET scans were initiated 1 h after the intravenous ^18^F-FDG injection (9.25 MBq/kg) and continued for 10 min for each of two bed positions on different days over 1.5 months. Three scans were performed prior to monkeypox virus inoculation (days −20, −15, and −5) and up to six scans were conducted postinoculation (days +1 or +2, +3 or +4, +7 or +8, +10, +16, and +21). The scans were conducted in the morning; the animals were fasted overnight for 12 h prior to the scanning session. The blood glucose concentrations were measured prior to the ^18^F-FDG injection before each scanning sessions. PET images were acquired in three-dimensional (3D) mode and reconstructed iteratively using 3D-ordered subsets expectation maximization algorithm with two iterations and nine subsets followed by 18 iterations of maximum *a posteriori* reconstruction [17]. Maximum *a posteriori* parameters were adjusted to provide a uniform spatial resolution of 1.8 mm (FWHM = 1.8 mm) in all three directions. Methods for scatter, decay, random, and attenuation correction were applied during the process of PET image reconstruction.

CT images were acquired with a CereTom® (NeuroLogica Corp., Danvers, MA, USA) 8-slice mobile head-and-neck CT scanner that was installed in close proximity to the microPET scanner. The CereTom® CT scanner provided 190 slices with 0.49 × 0.49 mm in-plane resolution and 1.25-mm slice thickness that were acquired at 120 kVp and 5 mA. CT scans were taken either immediately before or after PET imaging to ensure consistent animal position and fusion of the PET and CT scans for data analysis. Incorporating the use of the same table for both scanners eliminated the need for animal repositioning. To restrict animal motion, the animal was secured by anchoring the limbs and by controlling the level of anesthesia. CT scans were used for attenuation correction and coregistration with PET images to define anatomical localization of the LNs of interest. In addition, CT images were used to obtain the LN volume applied for SUV_mean_ computation to determine interscan data reproducibility prior to viral challenge.

### Image analysis

**Figure 1:**
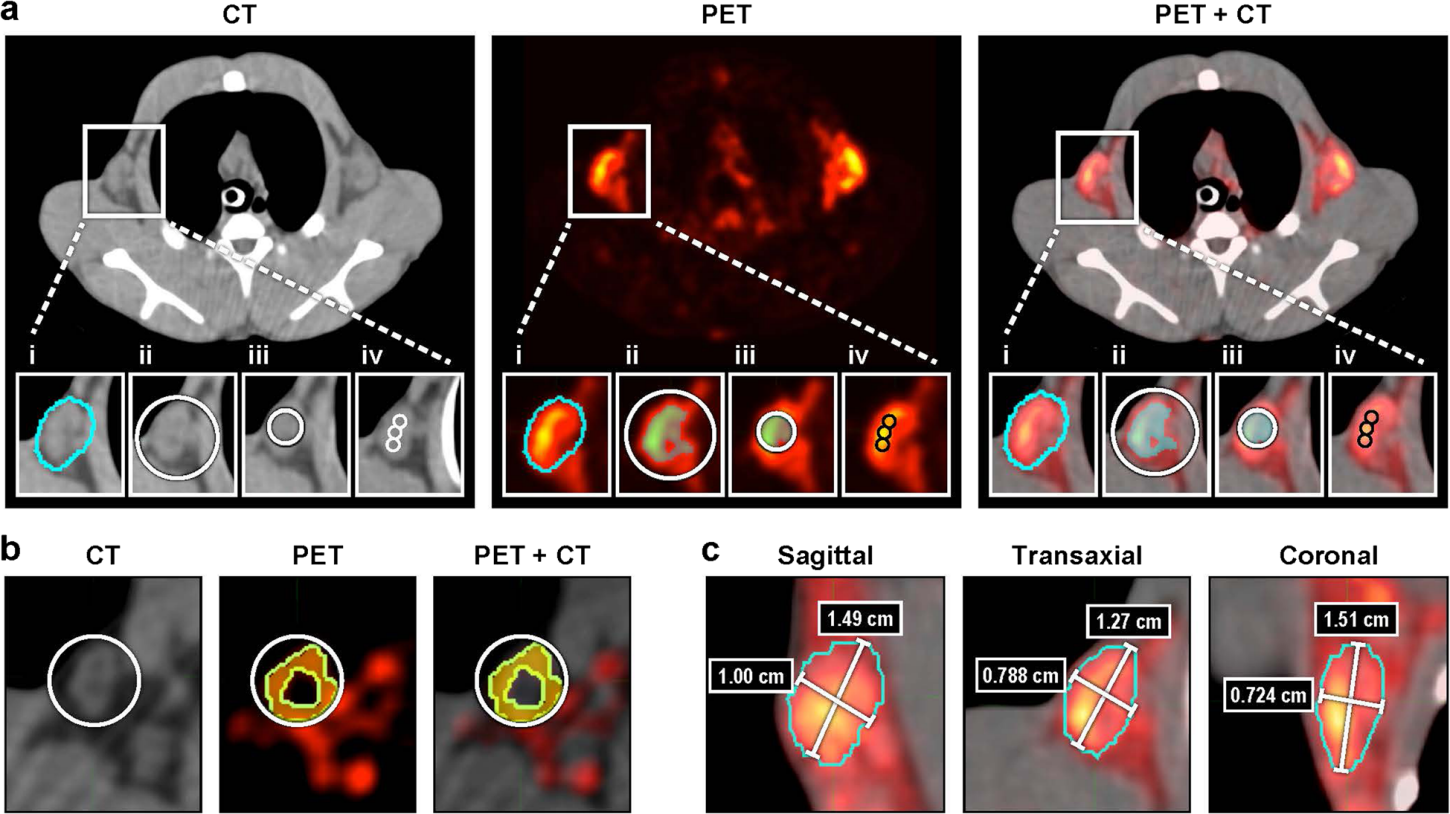
**Definition of ROIs used for** ^**18**^ **F-FDG uptake quantitation in the axillary LN.** CT, PET, and fused CT/PET images of the same LN obtained on day 3 **(b)** and day 8 **(a,c)** postinoculation of monkeypox virus. **(a)** SUV_mean_ (i), SUV_threshold_ (ii), mSUV_threshold_ (iii), and SUV_fixed volume_ (iv) (see “Methods” for details). **(b)** An example of inclusion of the voxels (chartreuse) from surrounding tissue with higher SUVs compared to the voxels (black) within the LN when SUV_threshold_ metrics was applied to PET image. **(c)** Delineation of a LN center and dimensions for VOIs placement within the LN edges. Images represent a middle slice of a LN in sagittal, transaxial, and coronal views showing the long and a short axis dimensions. The intersection of the two axes identifies a LN center.

Data analysis was performed using MIM workstation software version 5.2.2 (MIM Software Inc., Cleveland, OH, USA). The largest LN in the axillary fossa was chosen for analysis. This LN usually is positioned close to the body surface and easy to identify. VOIs were first specified on CT images coregistered with PET images. The volume of a whole LN was delineated by manually drawing the peripheral boundary on each of the slices where it appeared (Figure 1a (i)). SUVs from all the voxels inside this volume were averaged to calculate SUV_mean_. The SUV with the highest value within the LN volume identified by the MIM program was recorded as SUV_max_.

To compute the SUV_threshold_, a ‘threshold’ option in MIM was selected to place a sphere around the whole LN (Figure 1a (ii)). The tool averaged the voxels inside the sphere above a specified threshold that was set at 50% of the maximal voxel value. As the LN metabolic activity was often below that of surrounding tissue at baseline (Additional file 2) and during early infection (day 3/4, Additional files 2 and 3, Figure 1b), we also used a modified SUV_threshold_ (mSUV_threshold_) method to exclude the voxels outside the LN. mSUV_threshold_ was calculated by averaging all the voxels with SUVs above 50% of the maximal value within a sphere inside the LN edges (Figure 1a (iii)). The diameter of a sphere was chosen based on the smallest LN axis defined on CT images. First, to specify the smallest axis, the CT images in 3D view were examined to identify the edges and the center of a LN (Figure 1c). When the approximate center of a LN was located, two perpendicular lines were drawn on LN slices in transaxial, coronal, and sagittal views following the largest and the smallest LN axis on each view (Figure 1c). When the sphere diameter was specified, the sphere was placed strictly within the edges of the LN (Figure 1a (iii)).

We also applied a fixed dimension method, SUV_fixed volume_, by creating a template of three identical small spheres (0.2 cm diameter, total of 21 full voxels in all three spheres), placed contiguously within the longest axis of the LN. The sphere diameter was chosen based on the smallest LN axis (range 0.25 to 0.39 cm) among all animals determined on baseline CT images. The three spheres were transferred to each new data set by determining the center of the LN (defined by the intersection of axes in 3D view, Figure 1c), placing the middle sphere in the LN center and the other two spheres adjacent to the first one along the long axis of a LN in transaxial view. By using three small spherical VOIs, we adjusted the VOI location to the shape of the LN of each subject. Although the size and shape of the LNs differed, the VOI covered similar locations in the middle of each LN. The SUV_fixed volume_ was computed by averaging the SUVs from 21 voxels covered by three spheres.

For SUV calculation, the radioactivity concentration from the VOI on the PET image was divided by the injected dose and normalized to the body weight of the animal and radioactive decay for the time point of ^18^F-FDG injection

$$ SUV = decay\  corrected\  radioactivity\  concentration\ \left( MBq/ ml\right)\kern0.5em \div injected\  dose\ (MBq)\div body\  weight\ (g) $$

### Statistical analysis

The correlation between the volume measurement on pre-inoculation CT scans 1 and 2, 1 and 3, and 2 and 3 was calculated using the Pearson product-moment correlation coefficient (*r*). Application of the Kolmogorov-Smirnov test [18] confirmed that the difference between the pairs of scans followed a Gaussian distribution. To investigate the interscan reproducibility of three baseline scans, we compared scans 1 and 2, 1 and 3, and 2 and 3 for LN volumes and SUV_mean_, SUV_max_, SUV_threshold_, mSUV_threshold_, and SUV_fixed volume_ using Bland-Altman analysis [19]. The mean difference, standard deviation of the mean differences (SD), coefficient of repeatability (CR), and limits of agreement (LoA) were calculated and represented as Bland-Altman plots. The SD was calculated by squaring all the differences, adding them up, dividing them by the number of measurements, and taking the square root. The LoA were calculated by adding (upper limit) or subtracting (lower limit) the CR, defined as CR = 1.96 × SD, from the mean difference. This analysis of data reproducibility was performed with the assumption that the animal health status did not change during the time the scans were obtained 5 to 21 days prior to monkeypox virus inoculation. Unchanged animal health status was confirmed by physical examination.

Bland-Altman analysis was subsequently used to investigate the agreement between five VOI metrics for peak ^18^F-FDG uptake in the LNs of survivors on day 10 after inoculation. The differences obtained for each animal were plotted against the mean differences of the respective pairs of VOI measures. For acceptable agreement, the 95% LoA (±1.96 SD of the mean difference) should include 95% of the difference between the methods of measurement. Two-way repeated measures analysis of variance (ANOVA) was employed to explore the difference between treated and untreated groups or surviving and moribund groups in LN ^18^F-FDG uptake using SUVs with five different VOI metrics. We used ^18^F-FDG uptake value at different time points (days −1, −2, −3 pre- and days +1 or +2 and +3 or +4 postinoculation with monkeypox virus) as within factor and treatment or disease outcome as between factors, respectively. *Post hoc* comparisons were performed using Bonferroni test. GraphPad Prism 6.01 (GraphPad Software Inc., La Jolla, CA, USA) was used for all statistical analyses.

## Results

**Figure 2:**
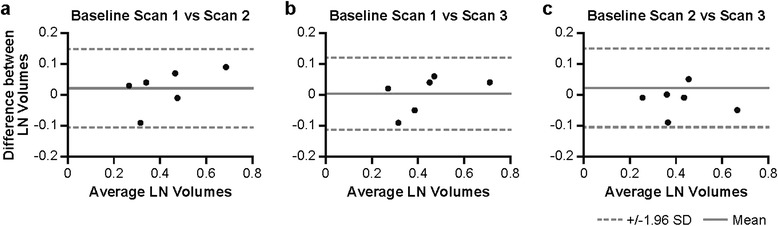
**Bland-Altman plots comparing the agreement between the LN volume measurements from baseline CT scans.** Scans were performed on three separate days prior to monkeypox virus inoculation. Differences between two pairwise measurements of LN volumes in each animal (*n* = 6) (scans 1 and 2 **(a)**, 1 and 3 **(b)**, and 2 and 3 **(c)**) are plotted against mean volume of the two scans. Dashed lines show the limits of agreement, and solid lines show the mean difference between LN volumes.

**Figure 3:**
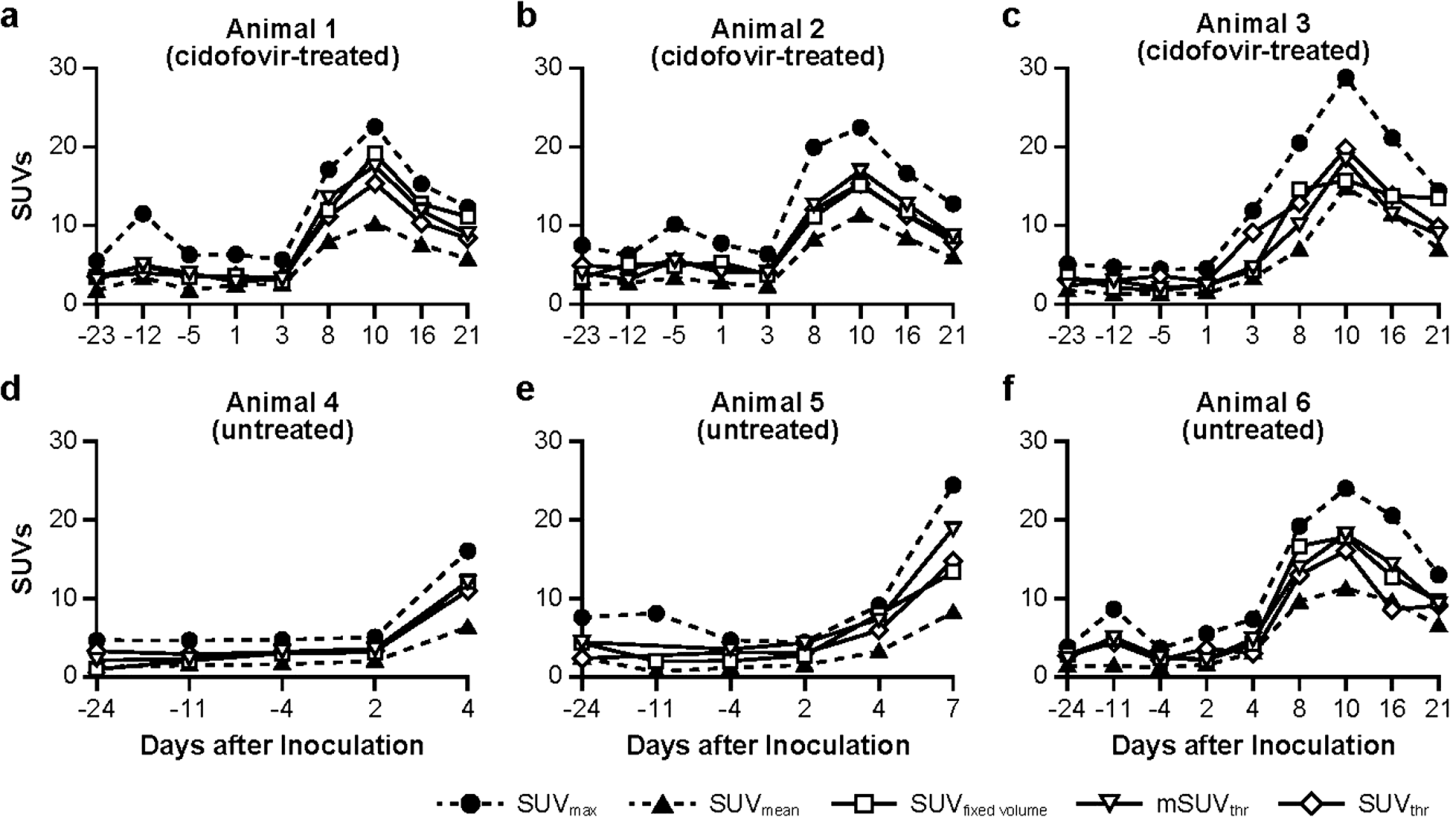
**Impact of VOI definition on the SUVs of axillary LN during monkeypox virus infection.** Animal numbers 1 to 3 **(** **(a)**, **(b)**, **(c)** **)** and numbers 4 to 6 **(** **(d)**, **(e)**, **(f)** **)** represent those treated with cidofovir and those left untreated, respectively. Animal numbers 1 to 3 and number 6 survived the infection.

Three baseline CT scans prior to monkeypox virus inoculation were used to investigate interscan repeatability of analysis of LN volumes from the right axilla for application in SUV_mean_ computations. The mean LN volume and SD averaged for all six subjects from pre-inoculation scans 1, 2, and 3 were 0.43 ± 0.17, 0.42 ± 0.14, and 0.43 ± 0.14 cm^3^, respectively, as measured on CT images. The correlation coefficient, *r,* of pairwise comparisons between the LN volume determined on baseline scans 1 and 2, 1 and 3, and 2 and 3 was 0.94 (*p* = 0.006), 0.95 (*p* = 0.004), and 0.94 (*p* = 0.005), respectively. A good agreement between the LN volume measurements on different days before inoculation was confirmed by Bland-Altman analysis (Figure 2). The mean differences and SD of the LN volume from scans 1 and 2 (Figure 2a), 1 and 3 (Figure 2b), and 2 and 3 (Figure 2c) were 0.022 ± 0.06, 0.003 ± 0.060, and 0.018 ± 0.048 cm^3^, respectively. CRs of these scans were 0.13, 0.12, and 0.09, respectively.

The range of blood glucose concentrations for all animals (*n* = 6) measured before each scanning session varied between 60 and 72 mg/dL. No correlation was noted between changes in ^18^F-FDG uptake over the course of monkeypox virus infection and the variation in glucose concentration measured before each scanning session on different days before and after virus inoculation.

Mean SUV ± SDs for three baseline scans, 1.98 ± 0.74 (SUV_mean_), 6.25 ± 2.21 (SUV_max_), 3.55 ± 0.90 (SUV_threshold_), 3.36 ± 1.09 (mSUV_threshold_), and 3.20 ± 1.25 (SUV_fixed volume_) in all the animals varied depending on VOI metrics. To determine interscan variability, the mean differences in SUVs from each of the three baseline PET scans were calculated by Bland-Altman analysis. Although all the differences for the SUVs were within ±1.96 SD, the worst agreement for the baseline scans was with SUV_max_ as shown by greater values for three outcome measures of Bland-Altman analysis, the mean difference and SD, 95% LoA, and CR (Table 1). Compared with mSUV_threshold_ and SUV_fixed volume_, SUV_mean_ and SUV_threshold_ had smaller SDs on different scanning days as a result of larger volumes used in these SUV computations.

**Table 1:** **Repeatability of** ^**18**^ **F-FDG SUV measurements in the LN of normal rhesus macaques using SUVs with different VOI metrics**

**SUV method scan pair comparison**	**Mean difference ± SD**	**95% limits of agreement: lower limit, upper limit**	**Coefficient of repeatability**
SUV_fixed volume_			
Scans 1 and 2	−0.40 ± 1.84	−4.01, 3.20	3.60
Scans 1 and 3	0.16 ± 1.74	−3.25, 3.57	3.41
Scans 2 and 3	0.57 ± 1.18	−1.75, 2.88	2.31
mSUV_threshold_			
Scans 1 and 2	−0.89 ± 1.42	−3.68, 1.90	2.79
Scans 1 and 3	−0.41 ± 0.92	−2.21, 1.39	1.80
Scans 2 and 3	0.22 ± 1.94	−3.59, 4.03	3.81
SUV_threshold_			
Scans 1 and 2	−0.28 ± 0.89	−2.02, 1.46	1.74
Scans 1 and 3	−0.21 ± 0.50	−1.19, 0.78	0.99
Scans 2 and 3	0.19 ± 1.26	−2.28, 2.65	2.47
SUV_mean_			
Scans 1 and 2	0.004 ± 1.08	−2.12, 2.19	2.12
Scans 1 and 3	0.15 ± 0.72	−1.26, 1.56	1.41
Scans 2 and 3	0.12 ± 0.78	−1.41, 1.64	1.52
SUV_max_			
Scans 1 and 2	−1.61 ± 3.01	−7.52, 4.29	5.91
Scans 1 and 3	0.004 ± 1.82	−3.52, 3.53	3.53
Scans 2 and 3	1.65 ± 3.51	−5.23, 8.54	6.88

Following inoculation of monkeypox virus, the characterization of infection in NHPs and histological evaluation of LN tissue during infection is described in Additional file 1. One animal in the untreated group survived the infection, while the remaining two subjects became moribund on day 7 after inoculation.

**Figure 4:**
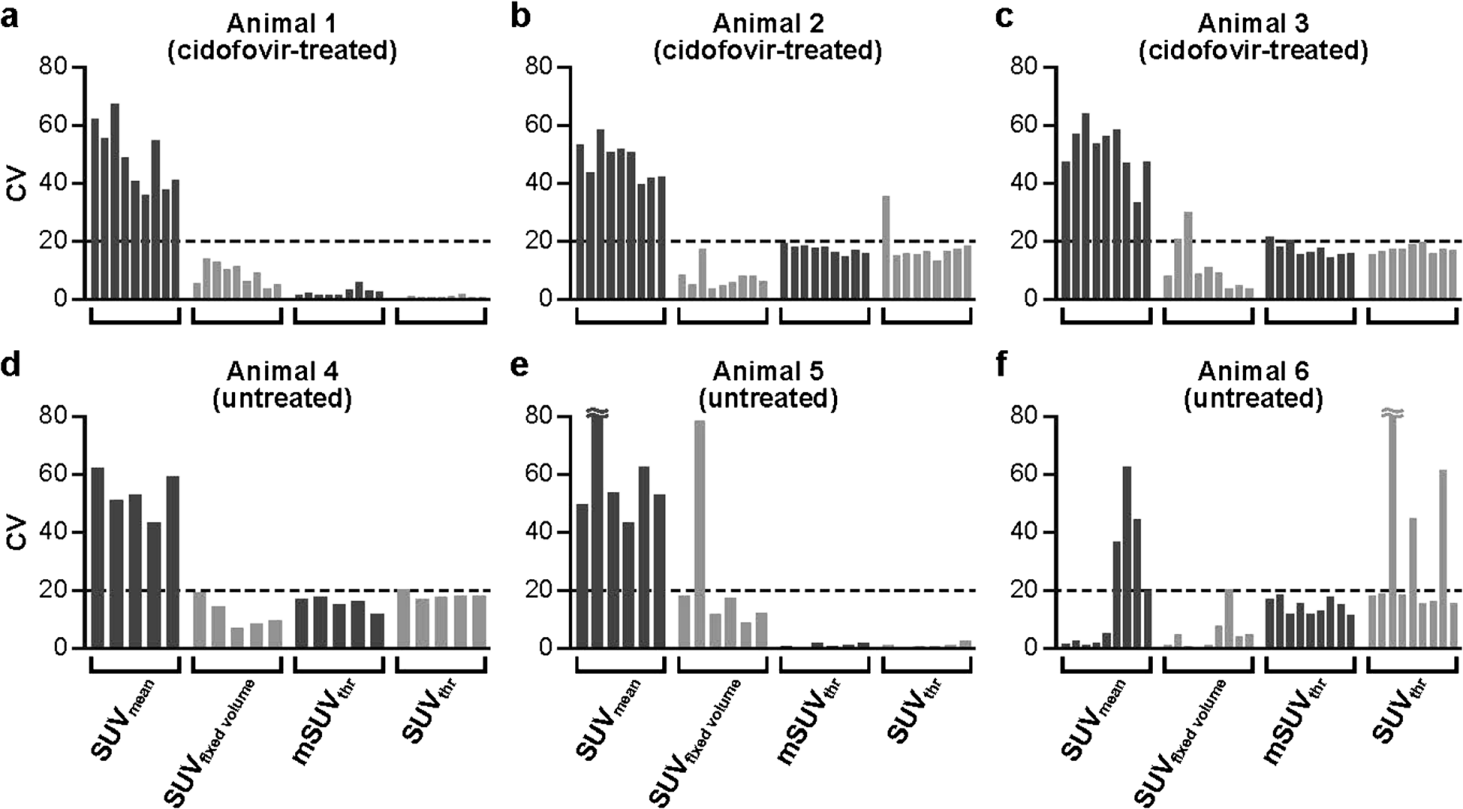
**CV for SUV**_**mean**_ **, SUV**_**fixed volume**_ **, mSUV**_**threshold**_ **, and SUV**_**threshold**_ **calculated for each scanning session in each animal.** Each column of four sets of data shown by different shades of black and gray represent CVs for a single time point before (pre-inoculation days −20, −15, and −5) and after (days +1 or +2, +3 or +4, +7 or +8, +10, +16, and +21) virus inoculation. On the x-axis, the infection progression is from the left to the right (e.g., first three columns from the left in each data set represent the data from three sequential baseline scans prior inoculation). Animal numbers 1 to 3 **(** **(a)**, **(b)**, **(c)** **)** and numbers 4 to 6 **(** **(d)**, **(e)**, **(f)** **) **represent those treated with cidofovir and those left untreated, respectively.

In examining VOI metrics, all five SUVs showed good concurrence for the pattern of changes in LN metabolic activity in survivors (animal numbers 1 to 3 [cidofovir-treated], number 6 [untreated]) over the course of monkeypox virus infection (Figure 3). ^18^F-FDG uptake was low from −5 days prior to inoculation (Additional file 3) through +3 days postinoculation (Additional files 2 and 3), increased markedly after day +3 postinoculation, and peaked on day +10 (Figure 3). Average SUVs ± SDs in all surviving animals on day +10 postinoculation were 12.02 ± 2.00 (SUV_mean_), 24.48 ± 3.01 (SUV_max_), 16.65 ± 2.14 (SUV_threshold_), 17.69 ± 0.69 (mSUV_threshold_), and 16.98 ± 1.84 (SUV_fixed volume_) (Figure 3a,b,c,f). Similar to the SUVs obtained from LNs prior to inoculation, the values were comparable among SUV_fixed volume_, mSUV_threshold_, and SUV_threshold_ but were always above SUV_mean_ and less than the SUV_max_ by 30% to 40%. In addition, SUV_mean_ for each of the animals were characterized by substantial SD as a result of great SUV variability of the voxels included in the calculation, which led to coefficients of variation (CV) for SUV_mean_ between 40% and 60% on most scanning days (Figure 4). In contrast, the CVs for SUV_fixed volume_, mSUV_threshold_, and SUV_threshold_ were rarely greater than 20%. These higher CV values were mostly observed on the images from the baseline scans with a low rate of LN metabolic activity that was comparable to surrounding tissue.

**Figure 5:**
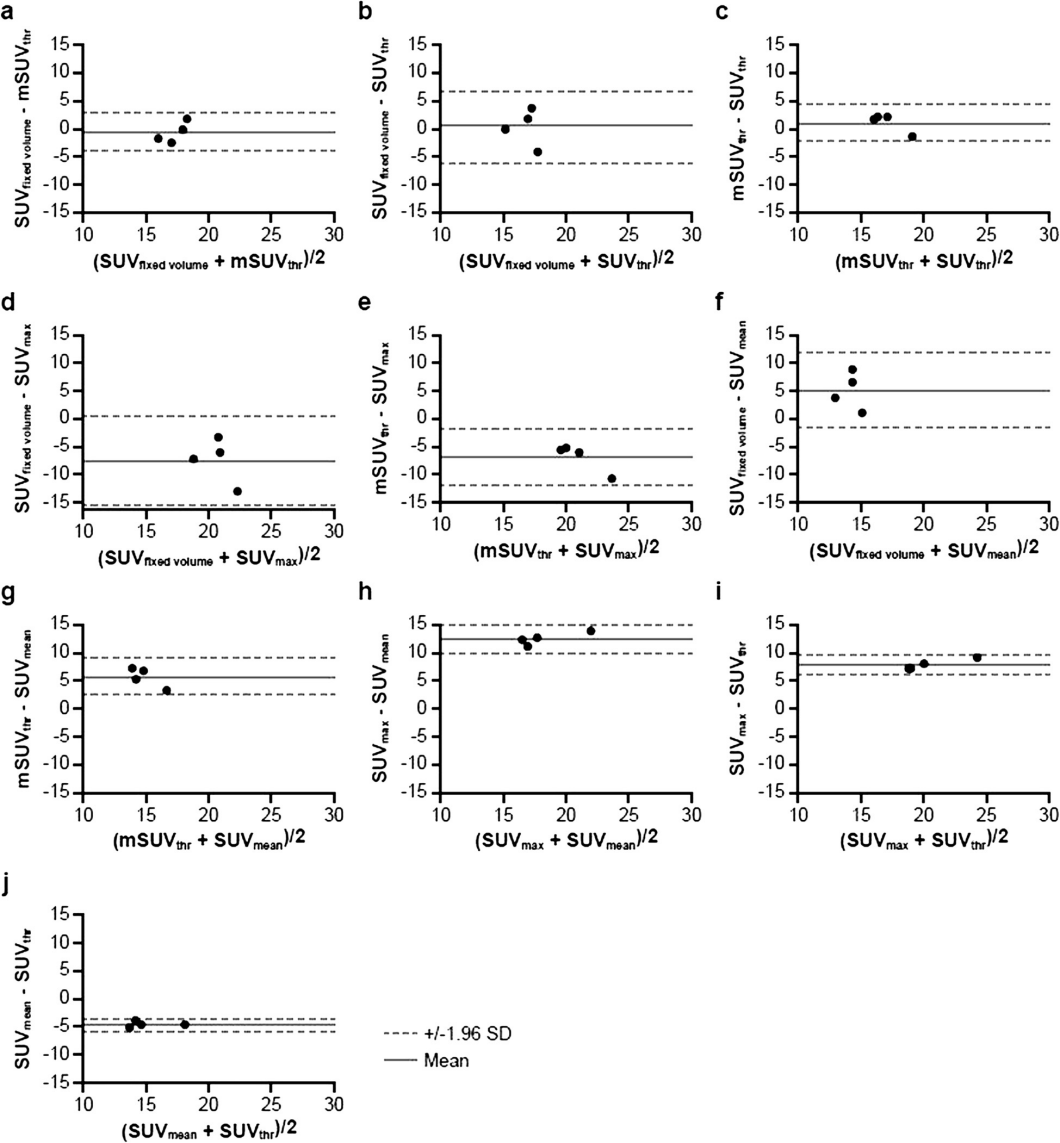
**Comparison of SUVs in LNs on day 10 postinoculation with monkeypox virus of survivors.** Pairwise comparisons were performed between SUVs with varied VOI metrics: **(a)** SUV_fixed volume_ vs mSUV_threshold_, **(b)** SUV_fixed volume_ vs SUV_threshold_, **(c)** mSUV_threshold_ vs SUV_threshold_, **(d)** SUV_fixed volume_ vs SUV_max_, **(e)** mSUV_threshold_ vs SUV_max_, **(f)** SUV_fixed volume_ vs SUV_mean_, **(g)** mSUV_threshold_ vs SUV_mean_, **(h)** SUV_max_ vs SUV_mean_, **(i)** SUV_max_ vs SUV_threshold_, and **(j)** SUV_mean_ vs SUV_threshold_ in four surviving animals. Dashed lines show the limits of agreement, and solid lines show the mean difference between LN volumes.

For the peak ^18^F-FDG uptake observed in survivors on day +10 postinoculation, the best correspondence was between SUV_fixed volume_ and mSUV_threshold_ (Figure 5a). The mean difference and SD between the two measurements were (−0.71) ± 1.84. Similar agreement was between mSUV_threshold_ and SUV_threshold_ with the bias of (1.043) ± 1.71 (Figure 5b) and between SUV_fixed volume_ and SUV_threshold_, but the SD was greater between the latter two SUV methods (Figure 5c). The Bland-Altman plot revealed poor agreement between SUV_fixed volume_ and SUV_max_, mSUV_threshold_ and SUV_max_, SUV_fixed volume_ and SUV_mean_, mSUV_threshold_ and SUV_mean_, and SUV_mean_ and SUV_threshold_ as shown by the mean difference between any of the two pairs of 5 or greater or less than −5 (Figure 5d,e,f,g,h). Very poor agreement was found between SUV_max_ and SUV_mean_ (Figure 5i) and between SUV_max_ and SUV_threshold_ (Figure 5j); the bias and SD between the two measurements were (12.5 ± 1.2) and (7.8 ± 0.89), respectively.

**Figure 6:**
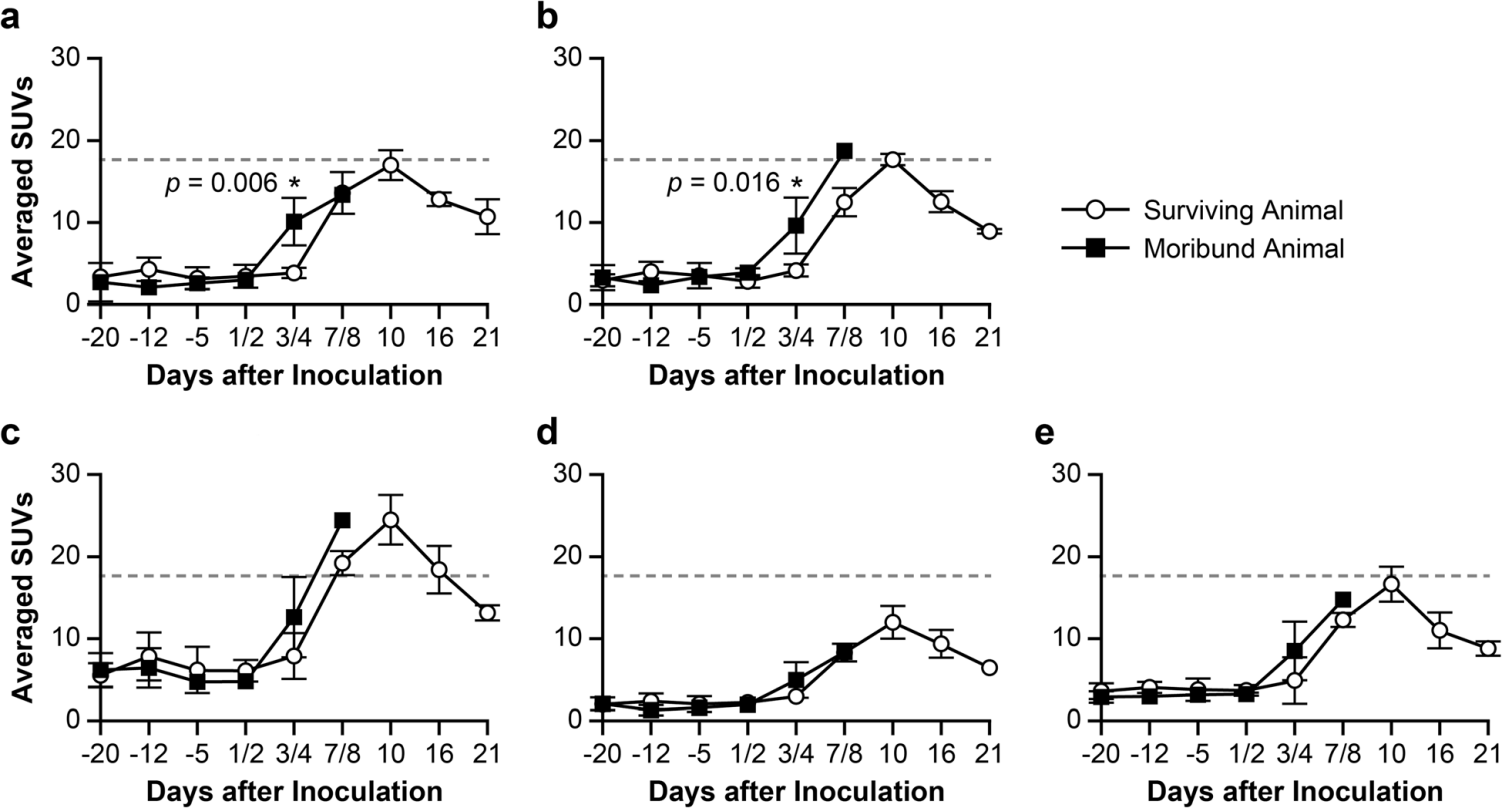
**SUVs in the axillary LN during monkeypox virus infection in surviving and moribund groups.** SUVs were assessed by five methods for VOI metrics, **(a)** SUV_fixed volume_, **(b)** mSUV_threshold_, **(c)** SUV_max_, **(d)** SUV_mean_, and **(e)** SUV_threshold_, in four surviving and two moribund animals.

PET images revealed an increase in ^18^F-FDG-PET uptake by the LNs on day +3 or +4 postinoculation in animals that eventually became moribund compared with surviving animals (Figure 6a and Additional file 3 show CT/PET images of one representative animal from each group). To explore whether the pattern of changes in a LN metabolic activity can predict a disease outcome and specify the effect of cidofovir treatment, we analyzed a subset of the data up to day +7 postvirus inoculation by two-way repeated measure ANOVA. Results of statistical analysis indicate differences in the pattern of ^18^F-FDG-PET uptake changes in moribund and surviving groups at early time points postinoculation (up to day +3 or +4) with SUV_fixed volume_ and mSUV_threshold_ only (statistically significant interaction between moribund and surviving groups, *p* = 0.006 and *p* = 0.0164, respectively, Figure 6b,c). Other SUV methods did not distinguish between changes in ^18^F-FDG-PET uptake in moribund and surviving groups (Figure 6d,e,f). Bonferroni’s multiple comparison test specified a statistically significant increase in ^18^F-FDG uptake in moribund group on days +3 or +4 postinoculation compared with surviving group (adjusted *p* = 0.0037 and *p* = 0.0001, for mSUV_threshold_ and SUV_fixed volume,_ respectively). No statistically significant effects were found between cidofovir-treated and untreated groups.

## Discussion

In this paper, we compared the agreement between conventional and modified VOI metrics to identify which metric(s) provide(s) reproducible SUVs when monitoring ^18^F-FDG uptake in the LN over the course of monkeypox virus infection (Table 2). The interscan data reproducibility for the LN volume in baseline scans ensures consistency in defining the whole LN boundaries on CT images and indicates the degree of reliability of VOI for SUV_mean_ calculation. Among five methods for the SUV calculation, SUV_max_ demonstrates the worst agreement between three baseline scans. These results confirm findings from similar studies involving cancer patients [15,20]. Replicate scans performed within a short period of time on the same patient using an identical technique often produce poorer interstudy reproducibility for SUV_max_ than for SUVs averaging greater volumes [15]. Taking into account the low baseline rate of LN metabolic activity that was comparable to the background rate in surrounding tissue and small LN size, the repeatability of SUV measurement for the baseline scans in our study is improved by increasing the volume included in other VOI metrics.

**Table 2:** **Advantages and drawbacks of SUV methods with different VOI metrics used in the current study** [5-8,11-15,21,22]

**SUV metrics**	**Current use in clinical settings**	**Major drawbacks or advantages**	**References**	**Statistically significant effects in current study**
SUV_mean_	+	• PVE at the edges of target region	[5,6,11-14,21]	No
		• Time consuming to outline target region		
		• Inclusion of nontarget tissue		
SUV_max_	+	• Susceptible to noise	[8,21,22]	No
		• Random voxel location		
		• Independent of observer		
		• Easy to apply		
SUV_threshold_	+	• Inclusion of nontarget tissue	[7,14,15]	No
		• Less sensitive to noise		
		• Easy to apply		
		• More reproducible than SUV_mean_		
SUV_fixed volume_ (placement adjusted in current study)	+	• Less sensitive to noise than SUV_max_	[7,8]	Yes
		• Limits PVE at edges of target region		
		• Relatively similar location of voxels between scans and between animals		
mSUV_threshold_	−	• Less sensitive to noise than SUV_max_		Yes
		• Relatively easy to apply		
		• Limits PVE at edges of target region		

Overall, the qualitative pattern of changes in LN ^18^F-FDG uptake over the course of monkeypox virus infection is similar between SUVs with the five VOI metrics evaluated in this study (Figure 3). However, SUVs vary substantially depending on VOI definition. The SUVs obtained with SUV_threshold,_ mSUV_threshold_, and SUV_fixed volume_ VOI metrics are within similar ranges and always above the range for the SUV_mean_ and below the SUV_max_. SUV_max_ could be the most attractive method to use for monitoring an immune response using multiple sequential PET scans because SUV_max_ is independent of the observer and simple to apply, Table 2 [10,14,23]. Despite these advantageous properties, the use of SUV_max_ is greatly influenced by adverse effects of noise [21]. This weakness of SUV_max_ in combination with a random voxel location limit the ability of SUV_max_ not only to reproduce the measurement under normal conditions (baseline scans) but also to quantify reliably real changes in the LN metabolic activity during viral infection.

The major drawback of SUV_mean_ is the time consuming process of manual LN delineation on each of the slices in the 3D CT images set. Similar to small tumors, LN PET images are difficult to align perfectly with CT images that often have poor soft tissue contrast. As a result, individual SUV_mean_ during infection could be underestimated by inclusion of voxels with lower metabolic activity from surrounding tissue when manually defining the LN boundaries on CT images (Figure 6e). Partial-volume effect (PVE) at the edges is another factor contributing to underestimation of the values with SUV_mean_ [5,22,24]. Consequently, SUV_mean_ is characterized by substantial data variability within the VOI as shown by CVs generally greater than 40% (Figure 4). Similarly, the dynamic range of the SUV_mean_ is the widest compared with other methods (see Additional file 4).

The use of threshold-based methodology is attractive since the LN delineation on ^18^FDG-PET images is easy to perform, and this method provides more reproducible measures than SUV_max_ and SUV_mean_ [7,14]. A disadvantage of the threshold technique is that the threshold is chosen rather arbitrarily, and only metabolically active tissue can be used for its application. Setting a threshold in normal LNs is more difficult compared with active LNs as metabolic activity in normal LNs appears to be similar to that in surrounding tissue (Additional files 2 and 5). At early time points postinoculation, the ^18^F-FDG uptake in surrounding tissue is often higher than that in the LN leading to an inclusion of the majority of the voxels outside the LN edges in SUV_threshold_ computation (Figure 1b, Additional file 2). Similarly, this method proved to be unsuitable to monitor tumor response after treatment since nontumor tissue is very often also included in the VOI [14]. To eliminate inclusion of voxels from surrounding tissue with high ^18^F-FDG uptake, we limited the volume for thresholding by placing a spherical VOI in the middle of a LN. This modification in the procedure improves data variability as shown in Figure 4.

SUV_fixed volume_ focuses on the metabolic response within a restricted location of the LN taking into account changes in LN shape and size in rhesus macaques over the course of infection. This VOI metric includes selected voxels from a slice in the middle of a LN, where metabolic activity is usually higher compared with the LN edges, and covers relatively similar locations standardized between different animals and scanning sessions. Thus, by avoiding the edges of the LN, we minimize the PVE associated with SUV_mean_ metrics, and, similar to mSUV_threshold_, SUV_fixed volume_ excludes voxels from tissues surrounding the LN. SUV_fixed volume_ and mSUV_threshold_ demonstrate the best agreement among other SUVs between the values for the peak response in survivors (Figure 6). These two methods are associated with similar statistically significant increases in ^18^F-FDG uptake in moribund group at day +3 or +4 postinoculation of monkeypox virus compared with surviving group.

There are several limitations in our present work. Although the size of the LNs assessed in this study was within the scanner resolution, PVE correction was not applied. The best method to correct for PVE has yet to be determined as such correction by itself can produce a bias in measured uptake. Further studies are needed to explore the relevance of PVE correction in the context of PET imaging of the LN immune response.

The aim of this preliminary study in a limited number of subjects is to compare different methods for determining the SUV and choose the method(s) with the best agreement to be applied in future characterization of a LN response to viral infection. The low number of subjects is not sufficient to demonstrate whether other more subtle effects (e.g., treatment effects), besides differentiating between disease outcomes, can be demonstrated from application of the SUV_fixed volume_ and mSUV_threshold_ metrics. The current data do not provide any explanation for the lack of difference between treated and untreated survivors in terms of ^18^FDG uptake by the LN. Perhaps the lack of difference may be related to the sensitivity of the methods or the contribution of other processes not associated with metabolic activity.

Also, we cannot rule out the possibility that differences at day +3 or +4 postvirus inoculation could be explained by a suboptimal study design. To ensure consistency in viral stock properties (e.g., titers, number of passages) and in inoculation procedures, all NHPs were infected on the same day (day 0). However, imaging of treated and untreated groups was staggered over 2 days (e.g., days +3 or +4 postinoculation) to accommodate PET scanner availability and duration of pre- and postscan procedures in sick animals in a biosafety level 3 environment. Despite these limitations, a similar pattern of changes in ^18^F-FDG uptake in the LNs is observed in treated and untreated surviving NHPs irrespective of timing of scans but is not observed between untreated surviving and moribund animals scanned on the same day. Future studies with larger group of animals and improved study design will be able to clarify this issue. In addition, an intra- and interrater reliability evaluation of LN volume and SUVs should be considered in further studies. Another point for potential criticism for the current study could be the lack of ground truth for LN ^18^F-FDG uptake.

## Conclusions

We confirmed results of previous studies that quantification of changes in ^18^F-FDG-PET is highly sensitive to the method applied for PET image analysis. Evaluation of multiple approaches is necessary in choosing appropriate method(s) to monitor changes in LN metabolic activity during progression of infection. Results of our study indicate that SUV_fixed volume_ and mSUV_threshold_ are more reproducible than the other methods and provide the best agreement for SUV calculation. Both methods reduce the impact of noise, minimize the PVE, limit inclusion of background signals, and substantially decrease the SD of the mean SUVs. The improved precision of the SUV estimates with the proposed methods results in statistically significant difference between moribund and surviving groups at an early stage of monkeypox virus infection that is not detected with the other three methods. Therefore, SUV_fixed volume_ and mSUV_threshold_ are the preferred approaches rather than SUV_max_, SUV_threshold_, or SUV_mean_ for quantitative analysis of LN immune response over the course of monkeypox virus infection using ^18^F-FDG-PET. Consequently, these preferred methods may provide better tools for establishing ^18^F-FDG uptake by the LNs as a marker of functional response at an early stage of monkeypox virus infection.

## Additional files

Additional file 1:
**The macaque preparation, clinical course, and histological analyses following intravenous monkeypox virus challenge.**
Additional file 2:
**Elevated** ^**18**^ **F-FDG uptake in tissue surrounding the LN on day 3 after virus inoculation.** Maximum intensity projection (MIP) movie and representative ^18^F-FDG-PET images fused with CT images of an axillary LN in sagittal view acquired -5 days before and day +3 and 10 after virus inoculation. High ^18^F-FDG uptake is observed in tissue surrounding the LN on day +3 after virus inoculation. Additional file 3:
**Elevated** ^**18**^ **F-FDG uptake in axillary LN of an animal that eventually became moribund.** MIP movie and representative ^18^F-FDG-PET images fused with CT images of an axillary LN in sagittal view acquired before and on day 3 after monkey virus inoculation showing elevated ^18^F-FDG uptake in axillary LN of an animal that eventually became moribund. Additional file 4:
**Dynamic range for SUV**_**mean**_ **, SUV**_**fixed volume**_ **, mSUV**_**threshold**_ **, and SUV**_**threshold**_ **assessed before and after virus inoculation.** Dynamic range was calculated for single time points before (pre-inoculation days −20, −15, and −5) and after (days +1 or +2, +3 or +4, +7 or +8, +10, +16, and +21) virus inoculation in each animal. On the x-axis, the infection progression is from the left to the right. Data for voxels with negative values are not included. Additional file 5:
**Enlarged fused CT/PET images of axillary LN from representative moribund and surviving animals.** On day -5 pre- (top row) and day +3 or +4 postvirus (bottom row) inoculation.
