# Supplementary material for: The effect of volume of interest definition on quantification of lymph node immune response to a monkeypox virus infection assessed by 18F-FDG-PET
Source: EJNMMI Res. 2014 Sep 16;4:49. doi: 10.1186/s13550-014-0049-z (PMC4452685; doi:10.1186/s13550-014-0049-z)
Supplement: Additional file 1: — The macaque preparation, clinical course, and histological analyses following intravenous monkeypox virus challenge. [file 13550_2014_49_MOESM1_ESM.docx]

**Additional files**

**Title:** Comparison of different methods of ^18^F-FDG-PET image analysis for quantification of lymph node immune response to monkeypox virus infection

**Journal:** European Journal of Nuclear Medicine and Molecular Imaging

**Authors:** Svetlana Chefer • Christopher Z. Leyson • Jurgen Seidel • Reed F. Johnson • Joseph E. Blaney • Richard C. Reba • Peter B. Jahrling • Julie Dyall

**Corresponding author:** Svetlana Chefer, Integrated Research Facility, 8200 Research Plaza, Fort Detrick, Frederick Maryland, United States of America

Phone: (301)631-7323; Fax: (301)631-7389; E-mail:schefer@mail.nih.gov

**Additional file 1**

Description of Materials and Methods and Results describing the macaque preparation, clinical course, and histological analyses following intravenous monkeypox virus challenge.

Format: Docx. Size: 26 kb.

**Materials and Methods**

**Virus**

Monkeypox virus (MPXV) Zaire 79 strain (V-79-I-005) was propagated in Vero E6 cells at a multiplicity of infection of 0.1 for 7 days. Inoculum for challenge experiments was prepared by disruption of Vero E6 cells in an Ultrasonic processor VCX-750 (Sonics & Materials, Newton CT) for 120 seconds at 40% power on ice followed by centrifugation (500 x g, 10 min, 4˚C).

**Pre-enrollment nonhuman primate screening**

Upon screening, nonhuman primates (NHPs) were negative for simian retrovirus, simian T cell leukemia virus, vaccinia virus (VACV), cowpox virus, and MPXV and had no detectable antibodies against VACV as determined by flow cytometry against VACV-GFP [[30](#_ENREF_30)].

**Necropsy procedures**

Complete necropsies were performed after animals were humanely euthanized by established guidelines. Animals that became clinically ill and subsequently recovered were euthanized and necropsied at day 36 postexposure. Tissue samples were collected from all major organs for histopathological analysis and determination of viral load. Inguinal lymph node (LN) biopsies were taken on 2 days. On day -19/-20 before inoculation with virus, 1-2 right side inguinal LNs were collected from healthy subjects, and on day 9 postinoculation 1-2 LNs were collected from the left side in surviving NHPs, when we expected to see the strongest immune response in the LN based on the results from a previous study [[1](#_ENREF_1)]. Detailed results of immunological and histological tissue characterization are beyond the scope and limit of this manuscript and will be presented in a separate manuscript (unpublished work).

**Results**

**Characterization of monkeypox virus infection in NHPs**

All animals developed fever on day 1 or 2 postinoculation with monkeypox virus. Circulating antibody concentrations in surviving animals during recovery were the same in the three cidofovir-treated NHPs and the untreated survivor with a peak on day 16 postinoculation of monkeypox virus. The two moribund animals did not produce detectable concentrations of antibody before time of death (day 7). Treatment with cidofovir delayed skin lesion appearance and peak lesion count by 2 days and reduced lesion numbers by 5-10-fold. However, the moribund animals did not develop any skin lesions before their time of death. Similar to a previous study, treatment with cidofovir delayed viremia by 6 days and reduced peak viral titers by 2.4 logs in comparison to that observed in untreated animals [[31](#_ENREF_31)].

**Histological characterization of LN tissue**

On biopsy samples from baseline day -19, only limited B and T cell proliferation was detected in a few small follicles for both survivors and moribund animals. Coinciding with high ^18^F-FDG uptake seen in surviving animals on day 9 postinoculation, histological examination of inguinal LN biopsies showed marked histiocytosis with proliferating B cells in sinuses and T cell proliferation in the paracortex. Lymphoid depletion and necrosis was minimal. Recovering animals had normal LN architecture with strong follicular hyperplasia by day 22 and B cell proliferation in follicles. One of the moribund NHPs had LNs with severe lymphoid depletion and necrosis on day 7 at necropsy. Sinus histiocytosis with proliferating B cells was the most characteristic finding for survivors on day 9 and in both moribund animals on day 7 at time of necropsy.

**Additional file 2**

Enlarged fused CT/PET images of axillary LN in transaxial view. Images display the data from representative moribund and surviving animals on day 5 pre- (top row) and day 3/4 postvirus (bottom row) inoculation. Format: TIF. Size: 1MB.

**Additional file 3**

Movie of sequential ^18^F-FDG-PET scans performed on surviving animal 5 days before (a, d) and 3 (b, e) and 10 (c, f) days after monkeypox virus inoculation. Maximum intensity projection movies (a, b, c) and representative ^18^F-FDG-PET images fused with CT images of the right axillary LN in sagittal view (d, e, f). Low rate of metabolic activity is noticeable in the LN of interest marked by boxes on the movies (a, b) and by white arrow on the images (d, e). Increased metabolic activity in the tissue surrounding the LNs but not in the LN itself, is observed on day 3 post virus inoculation. Enlarged LN on day 10 post virus inoculation shows peak ^18^F-FDG uptake.

Format: MPEG-4 Size 5 MB.

**Additional file 4**

Maximum intensity projection movie and representative ^18^F-FDG-PET images fused with CT images of the right axilla in transaxial view acquired on day 3/4 postmonkeypox virus inoculation showing the difference in LN metabolic activity between an animal that eventually became moribund (a, c) and a surviving (b, d) animal.

Format: MPEG-4. Size: 4 MB.

**Additional file Resource 5**

Dynamic range (DR) for SUV_mean_, SUV_fixed volume_, mSUV_threshold_, and SUV_threshold_ was calculated for single time points before (pre-inoculation days -20, -15 and -5) and after (days 1 or 2, 3 or 4, 7 or 8, 10, 16 and 21) virus inoculation in each animal. On the x axis, the infection progression is from the left to the right. Data for voxels with negative values are not included.

Format TIF. Size: 432 kb.
