# Supplementary figures and images for: The effect of volume of interest definition on quantification of lymph node immune response to a monkeypox virus infection assessed by 18F-FDG-PET
Source: EJNMMI Res. 2014 Sep 16;4:49. doi: 10.1186/s13550-014-0049-z (PMC4452685; doi:10.1186/s13550-014-0049-z)

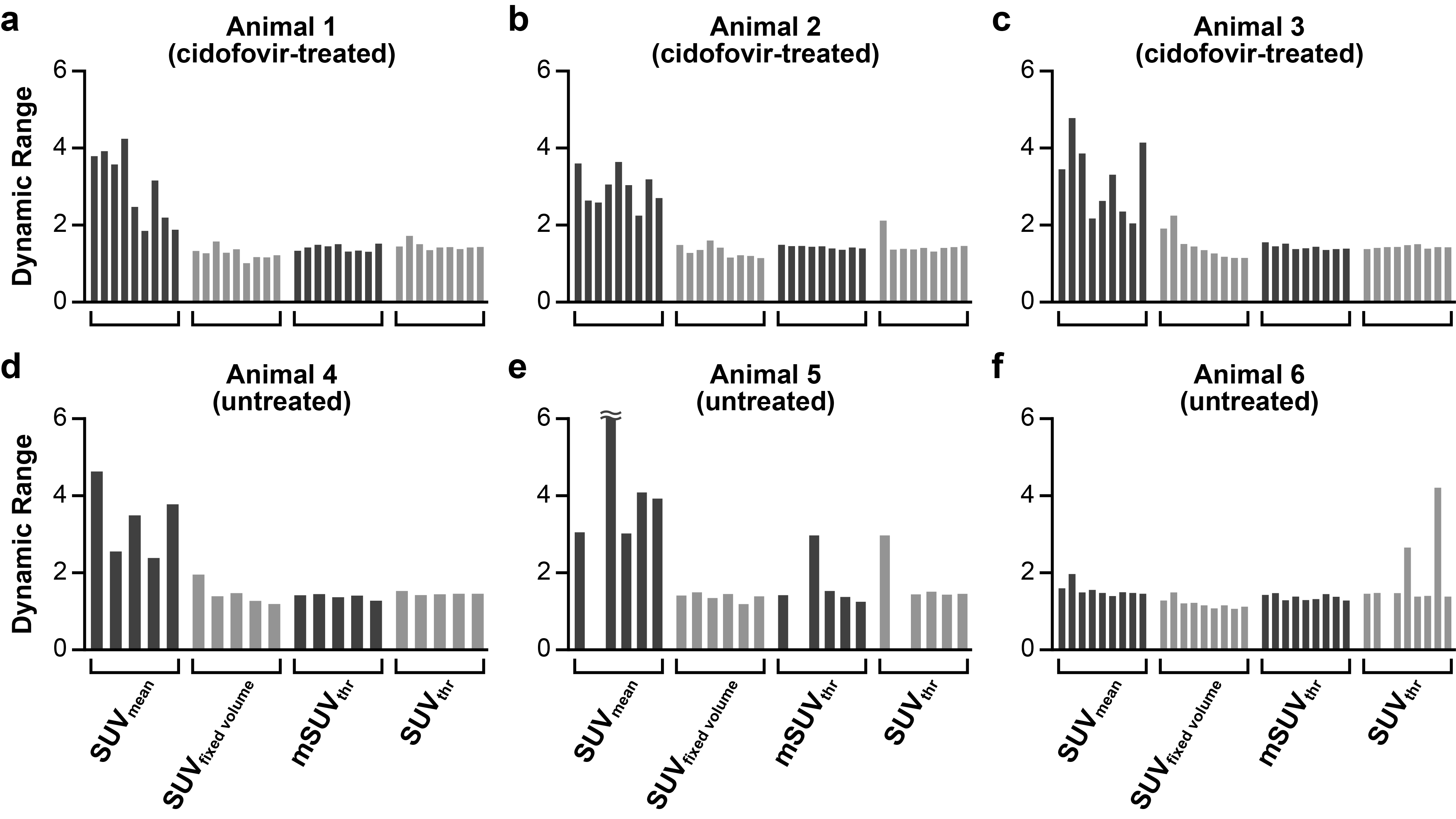

Supplement: Additional file 4: — Dynamic range for SUV mean , SUV fixed volume , mSUV threshold , and SUV threshold assessed before and after virus inoculation. Dynamic range was calculated for single time points before (pre-inoculation days −20, −15, and −5) and after (days +1 or +2, +3 or +4, +7 or +8, +10, +16, and +21) virus inoculation in each animal. On the x-axis, the infection progression is from the left to the right. Data for voxels with negative values are not included. [file 13550_2014_49_MOESM4_ESM.tiff]

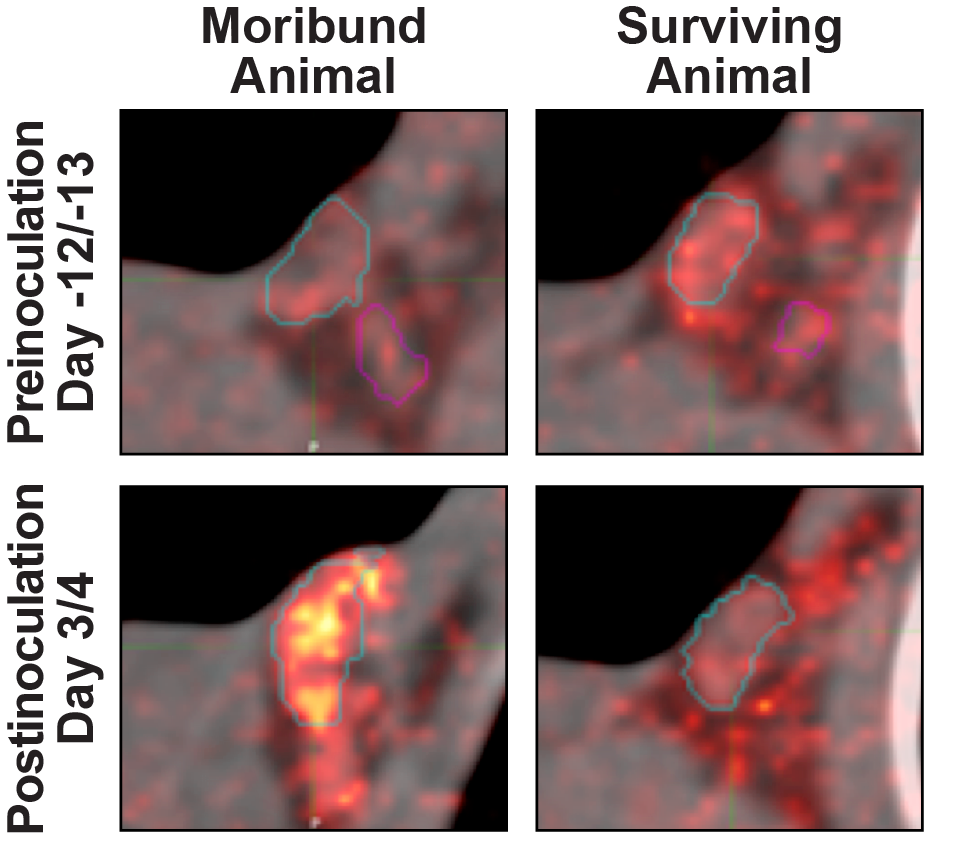

Supplement: Additional file 5: — Enlarged fused CT/PET images of axillary LN from representative moribund and surviving animals. On day -5 pre- (top row) and day +3 or +4 postvirus (bottom row) inoculation. [file 13550_2014_49_MOESM5_ESM.tiff]
